# Analysis of the wall thickness of intracranial aneurysms: Can computational fluid dynamics detect the translucent areas of saccular intracranial aneurysms and predict the rupture risk preoperatively?

**DOI:** 10.3389/fneur.2022.1075078

**Published:** 2023-01-09

**Authors:** Xin-xin Fan, Jie-wen Geng, Chuan He, Peng Hu, Li-yong Sun, Hong-qi Zhang

**Affiliations:** ^1^China International Neuroscience Institute (China-INI), Beijing, China; ^2^Department of Neurosurgery, Xuanwu Hospital, Capital Medical University, Beijing, China

**Keywords:** Polyflow, Ansys, CFD, translucent types, aneurysms, contour lines

## Abstract

**Background and purpose:**

The translucent area on the surface of intracranial aneurysms (IAs) is associated with rupture risk. In the present study, the Polyflow module of the Ansys software was used to simulate and analyze the thickness of the aneurysm wall to detect whether it was “translucent” and to assess the rupture risk.

**Methods:**

Forty-five patients with 48 IAs who underwent microsurgery were retrospectively reviewed. The medical records, radiographic data, and intraoperative images of the patients were collected. The image data were analyzed using computational fluid dynamics (CFD) simulations to explore the relationship between the simulated thickness of the aneurysm wall, the translucent area, and the rupture point of the real aneurysm's surface to predict the rupture risk and provide a certain reference basis for clinical treatment.

**Results:**

The Polyflow simulation revealed that the location of the minimum extreme point of the simulated aneurysm wall thickness was consistent with the translucent area or rupture point on the surface of the real aneurysm. There was a downward trend in the correlation between the change rate (I_S_) in the wall area and volume during aneurysm growth and rupture. Ruptured aneurysms have a greater inhomogeneity coefficient I_δ_ than the unruptured ones. In the unruptured group, translucent aneurysms also had greater inhomogeneity coefficients I_δ_ and more significant thickness changes (multiple I_BA_) than non-translucent ones.

**Conclusions:**

The Ansys software Polyflow module could detect whether the unruptured aneurysms were translucent and predict the rupture risk and rupture point.

**Clinical trial registration:**

https://clinicaltrials.gov/, Identifier, NCT03133624.

## Introduction

The prevalence of unruptured intracranial aneurysms (UIAs) is ~3–5% in the total population (1). However, the annual rupture rate of UIAs is estimated to be only 0.25%, and the majority of UIAs will never rupture (2). Due to the complexity of the natural history and diversity of UIAs, there is no unified scientific conclusion on the surgical indications for UIAs. It has been recognized that UIAs with a high rupture risk should be actively treated. Therefore, it is necessary to make a multidimensional prediction of the rupture risk of an aneurysm (3). The inflammatory reaction of the aneurysm wall is an important decisive factor in the formation, development, and rupture of intracranial aneurysms (IAs). Inflammatory cells infiltrate the aneurysm wall and release substances that destroy the structure. When the repair of the aneurysm wall fails to resist the inflammatory damage, the aneurysm grows, gradually resulting in a thinner wall. This process, coupled with the continuous impact of blood flow, will lead to rupture of the aneurysm wall and result in a subarachnoid hemorrhage (SAH). The aneurysm wall is a highly variable region containing areas of thick, intermediate, and super thin translucent tissues, each of which is distinguishable and quantifiable *via* intraoperative observation. In other words, intraoperative observation is the only effective way to confirm whether there is a translucent area on the surface of the aneurysm and the location of the rupture point. Along with an increase in size during the aneurysm's growth, the wall becomes thinner and translucent. When the thickness of these translucent areas becomes too thin to resist the pressure in the lumen, the aneurysm ruptures (4); this is also the underlying cause of rupture in most saccular aneurysms. Translucent aneurysms are more prone to rupture. The risk of rupture can be predicted to a certain extent by investigating the change in the thickness of the aneurysm wall. However, it is challenging to directly measure or investigate the thickness of the aneurysm wall, and there have been few studies performed in this area. Therefore, a simple, non-invasive, and effective method is needed to predict the thickness of the aneurysm wall. Polyflow, a module of the Ansys computational fluid dynamics (CFD) software based on finite element analysis, is primarily used for the flow simulation of elastic materials. Polyflow is suitable for solving the problems of extrusion molding, blow molding, wire drawing, and laminar mixing of plastics, resins, and other polymer materials, reducing the processing costs of polymer, glass, metal, and other materials. The present study aims to observe the translucent area or rupture point on the surface of true aneurysms to investigate the geometric parameters of the corresponding positions on the model surface. The concept of the “inhomogeneity coefficient” was proposed to verify whether Polyflow could effectively detect the translucent area or rupture point on the surface of aneurysms and therefore predict the rupture risk.

## Methods

Between January 2020 and September 2020, 66 consecutive patients with IAs who underwent microsurgical treatments at the Xuanwu Hospital were retrospectively analyzed. Simultaneously, the medical records, radiographic data, and intraoperative images of the patients were collected. The Institutional Review Board of Xuanwu Hospital approved the present retrospective study, and informed consent was waived in compliance with the Accountability Act.

### Ethics approval

The Institutional Ethics Committee of Xuanwu Hospital approved the present study (No. 2017082).

### Source of the data and imaging

In the present study, three-dimensional (3D) rotational angiography was performed using a 3131 IQ scanner (GE Healthcare, Buc, France). Rotational angiographic images were obtained during a 200-degree rotation with imaging conducted at 30 frames per second for a total of 5 s. The corresponding 510 projection images were reconstructed into a 3D data set of 512 × 512 × 512 voxels covering a field of view of 116 mm on a dedicated GE workstation.

### Quality control and data screening

**Figure 1:**
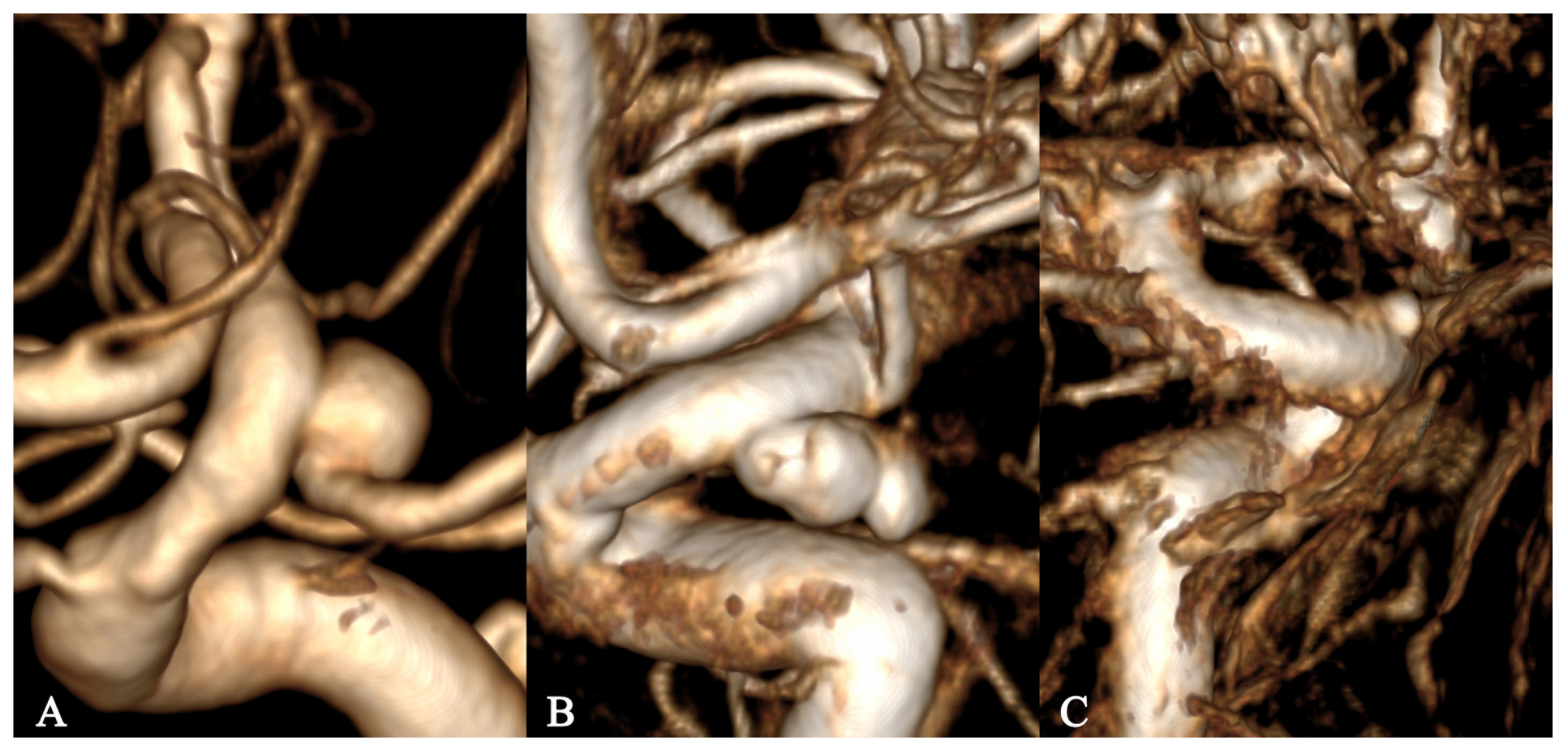
Grading of 3D image quality. **(A–C)** represent excellent, good, and poor grades, respectively.

The 3D image quality was divided into three levels, excellent, good, and poor, to acquire accurate research results from the perspective of CFD (Figure 1). An excellent level indicated good image quality and contrast, without artifacts or hollowing, with well-preserved detailed features of the aneurysm surface, a clear boundary with adjacent vessels, and accurate calculation results. A good level indicated a slightly poor effect despite an easily recognized and interpreted 3D image, with artifacts or hollowing and insufficient display of the detailed features of the aneurysm surface, and adhesion with adjacent blood vessels, which would still have a slight impact on the calculation results after clipping. A poor level indicated serious artifacts or hollowing in many regions on the 3D image, with distorted vascular morphology, barely displayed detailed features of the aneurysm surface, and serious adhesion with adjacent vessels, which would affect the recognition and interpretation (even after clipping), the morphology of the aneurysm, and losing the significance of CFD.

For the present study, the cross-sectional data of 45 patients with excellent vascular reconstruction quality were selected, including 48 aneurysms from 66 patients, of which 20 were ruptured.

### Surgical procedures and intraoperative videos

Patients undergoing microsurgery should be evaluated preoperatively by 3D rotational angiography, and microsurgery should be performed if a digital subtraction angiography (DSA) indicated difficulty in embolization or if the patients were not suitable for embolization. The standard pterion or Dolenc approaches were used in patients who underwent microsurgical treatments. All aneurysms had intraoperative confirmation of rupture points or translucent regions. All micromanipulations were conducted under a Pentero operating microscope (Carl Zeiss Pentero 800/900, Germany), with a video resolution of 1,920 × 1,080 pixels, and saved in the ^*^.mpg format.

According to a study of Kadasi et al. (4), aneurysms can be divided into the bright red, light red, and yellow categories with GNU Image Manipulation Program (GIMP 2.10.24, Free Software Foundation, Boston, Massachusetts, USA), which corresponded to the “semi-transparent area,” “medium-thickness area,” and “calcified area,” respectively.

### Surface model generation and segmentation

**Figure 2:**
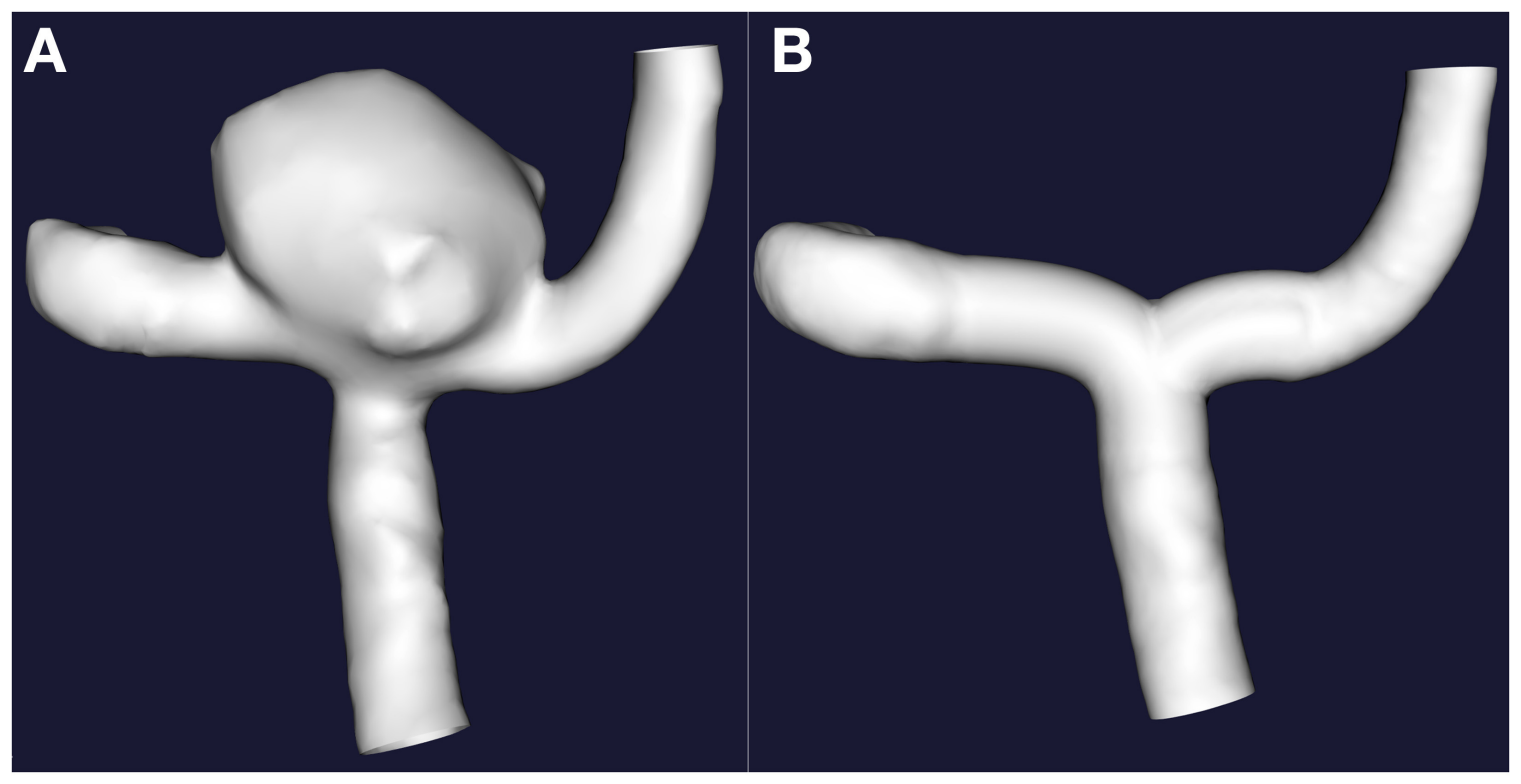
The interpolation method was implemented to remove the sacral cerebral aneurysm from its parent artery. **(A)** Original 3D images. **(B)** Only with retention of the 3D images of the parent artery.

The cross-sectional data were imported into Materialize Mimics (Medical) V21.0, the redundant blood vessels were erased, and only the aneurysm and the adjacent segment of the parent artery were preserved. The 3D model was then exported into a binary stereolithography (STL) file, which was post-processed using the VMTK program, including smoothing of the vascular surface, interpolation reconstruction of the parent artery (5), and removal of the saccular aneurysm from the parent vasculature. Finally, the models containing the aneurysm (A.stl) and the reconstructed vessel model of the parent artery (B.stl) were generated, as shown in Figure 2.

### Morphological measurement

The aneurysm neck was regarded as a curved surface, and the open ports of Model A and Model B were closed, respectively. The aneurysm volume was calculated by subtracting the volumes of Model B from Model A. Through the Boolean operation, the aneurysm surface area (S_A_) of Model A and the aneurysm neck surface area (S_B_) of Model B were obtained, respectively.

### Computational fluid dynamics process

The non-uniform rational basis spline (NURBS) model generation function of Geomagic Wrap 2021 (3D Systems, South Carolina, USA) was adopted to convert the A.stl and B.stl models into ^*^.igs format files. The two files were simultaneously imported into the Fluid Flow (Polyflow) module of the ANSYS 2021 R2 (ANSYS, Inc., Canonsburg, PA, USA) software. Model A was defined as solid, Model B as fluid, and then the mesh was generated. The next step is to define the mold. During this process, we click “Create a new task” to create a “F.E.M. task” of type “Time-dependent problem.” Since there is a contact problem between the fluid and the mold, it is necessary to define the mold's contact surface before defining the fluid's subtask. Click “Define molds” → “Create a new mold” → “Mold with constant and uniform temperature,” and define the mold name as “A.” Then define the scope of the mold and click on “Domain of the mold.” Select the area of mold A. Finally, define the contact conditions. Click on “Contact conditions” and change the value to “Contact.” After the mold definition is complete, go to the step of creating subtasks. Click “Create a sub-task” and select “shell model: Gen-Newtonian isothermal.” The fluid name is defined as “B.” Then, set the range of fluid B, material properties, initial thickness, contact conditions, and flow boundary conditions (6, 7). Next, save and exit. Finally, click on “Solution.” In the post-processor, the number of isolines was set to 200. The minimum extreme point of the isolines was compared with the translucent area or rupture point on the real surface of the aneurysm. The consistency of the two indicators was observed, and the thickness at the minimum extreme point was recorded.

### Definition of the inhomogeneity coefficient

The aneurysm surface area of Model A was defined as S_A_ and the average thickness of the aneurysm wall as ${\bar{\delta }}_{\text{A}}$. The initial area of this part of the vessel wall (the aneurysm neck) where the aneurysm would be formed in Model B was defined as S_B_ and the initial thickness as δ_B_. Then the volume of this part of the vascular tissue V = S_B_ · δ_B_ without considering the repair and proliferation of the vascular wall, the surface area of the aneurysm after blow molding was S_B_' and S_B_' = S_A_. The average thickness of the aneurysm wall after blow molding was ${\bar{\delta }}_{\text{B}}$ and ${\bar{\delta }}_{\text{B}}$ = ${\bar{\delta }}_{\text{A}}$, then V = S_B_ · δ_B_ = S_A_ · ${\bar{\delta }}_{\text{A}}$ = S_A_ · ${\bar{\delta }}_{\text{B}}$. The multiplier of the surface area increase was I_S_ = S_A_ / S_B_, the ratio of an average thickness (${\bar{\delta }}_{\text{B}}$) of the aneurysm wall after blow molding to the extreme point thickness (δ_B_') was defined as the local inhomogeneity coefficient I_δ_, and I_δ_ = ${\bar{\delta }}_{\text{B}}$ / δ_B_' = δ_B_ / (${\bar{\delta }}_{\text{B}}$' · I_S_).

### Grouping and statistical analysis

The cases were divided into the rupture group (Group A), the translucent unruptured group (Group B), and the non-translucent unruptured group (Group C). Based on the original data in Table 1, SPSS 22.0 statistical analysis software was used to investigate whether the differences in the data among the groups were statistically significant, including the differences in geometric parameters, the CFD results, and the inhomogeneity coefficient. The measurement data were expressed as mean ± standard deviation ($\bar{X}$ ± SD). In addition, it was necessary to determine which results could be used as indicators to predict the rupture risk of an aneurysm. Due to exceptional cases, such as giant aneurysms, particularly narrow neck or long narrow aneurysms, a certain number of outliers would be generated. These aneurysms' volume and simulated thickness parameters are significantly different from those of other aneurysms. We are not sure whether these outliers affect our experimental results. Therefore, it was necessary to eliminate these outliers and conduct statistical analyses again. The independent sample *t*-test or Kolmogorov-Smirnov test were adopted for the comparison among the groups. The countable data were expressed as composition ratios, and the *X*^2^ test was adopted for comparison. Spearman's or Pearson correlation analyses were used to analyze the correlation between the wall area change rate and volume. *P* < 0.05 was considered statistically significant.

**Table 1:** Basic information and original data of parameters of 48 aneurysms.

**Group A (*****n*** = **20)**						**Group B (*****n*** = **17)**						**Group C (*****n*** = **11)**					
**Aneurysm** **No**.	**Location**	**I**_s_	**Volume** **(mm**3**)**	**I**_BA_	**I**_δ_	**Aneurysm** **No**.	**Location**	**I**_s_	**Volume (mm**3**)**	**I**_BA_	**I**_δ_	**Aneurysm** **No**.	**Location**	**I**_s_	**Volume** **(mm**3**)**	**I**_BA_	**I**_δ_
1	AcomA	4.0406732118	94.76	35.23238381	8.719434105	6	PcomA	3.2341095028	56.6	71.90187	22.23235577	7	MCA	2.2663847780	23.95	6.023658613	2.657826981
2	MCA	5.7488915760	135.14	44.95078594	7.819035261	15	MCA	8.9100364174	715.49	37.388	4.196166567	13	MCA	2.4047175667	21.37	4.421742621	1.838778359
3	MCA	5.0767045455	15.97	36.59767886	7.208944018	16	A1	3.3973365112	36.88	14.82702	4.364306632	14	PcomA	2.4430479183	21.05	3.200261034	1.309946076
4	MCA	3.3598160151	55.96	36.55615736	10.88040452	17	MCA	7.9636119609	948.61	34.48276	4.330039907	22	MCA	3.8987811427	34.17	10.1707995	2.608712602
5	MCA	5.1861500907	59.64	74.14983001	14.29766372	20	MCA	2.0468671840	48.8	8.115747	3.964960471	24	MCA	6.5481566162	356.89	11.59281271	1.770393317
8	MCA	1.2706231454	10.53	4.906688249	3.861639281	23	R_MCA	5.3452695506	266.31	23.29317	4.357717044	27	MCA	5.6933842239	210	20.37232153	3.578244630
9	MCA	2.1621225201	35.08	5.255146491	2.430549815	26	MCA	4.5883528390	84.63	17.37567	3.786908664	30	AcomA	2.0661456072	4.92	2.721671564	1.317269971
10	AcomA	4.0713882199	49.49	11.77295756	2.891632270	34	ICA	9.4167033665	1473.81	158.0838	16.78759859	31	ICA	16.8198177498	1784.64	27.85632782	1.656161097
11	AcomA	4.3982797173	99.49	46.12817926	10.48777755	35	AcomA	4.0099857592	96.1055	12.62762	3.149043391	32	PcomA	3.7390956383	106.54	4.830872032	1.291989427
12	MCA	5.0645315892	297.79	48.15276168	9.507841117	36	MCA	4.6488589001	85.24	12.1121	2.605392002	38	MCA	2.0897745429	4.62	3.918495144	1.875080332
18	MCA	2.6434923391	43.85	11.72072305	4.433802541	37	MCA	3.8770003182	153.22	15.87997	4.095942063	43	MCA	2.2780536246	31.69	3.741549139	1.642432425
19	AcomA	2.8428240509	68.09	21.22183611	7.465054371	40	MCA	3.1086777238	132.86	7.073861	2.275520806						
21	MCA	3.5958280061	68.24	10.20929024	2.839204273	42	MCA	2.8222266481	237.23	6.861145	2.431110515						
25	MCA	2.7343674676	32.18	6.454987332	2.360687584	44	MCA	4.5506687826	271.35	20.81763	4.574631125						
28	MCA	3.2346451759	63.05	33.94194816	10.49325237	45	ICA	17.6140714753	7410.21	58.09859	3.298419229						
29	PcomA	3.3163436588	129.35	71.10987073	21.44225028	46	MCA	3.4841815541	131.49	11.26017	3.231797156						
33	PcomA	2.3277777778	4.77	6.397712118	2.748420480	48	MCA	5.3065290569	254.12	19.03512	3.587112391						
39	MCA	5.7870502316	17.44	23.71897186	4.098628993												
41	MCA	2.1217420081	132.29	7.471530737	3.521413399												
47	MCA	3.0023348637	199.75	8.743103144	2.912101262												

## Results

### Basic information

In the present study, 48 aneurysms from 45 patients were regarded as 48 independent samples. The morphological parameters and CFD results are summarized in Table 1 (the basic data of patients with multiple aneurysms were recorded repeatedly). The statistical calculation results of relevant parameters of the ruptured and unruptured groups are shown in Table 2. The statistical calculation results of relevant parameters of translucent and non-translucent types of the unruptured group are demonstrated in Table 3.

**Table 2:** Statistical results of parameters related to ruptured and unruptured aneurysms.

	**Group A (*n* = 20)**	**Group B + C (*n* = 28)**	***P*-value**
Age (years)	54.95 ± 9.78	57.46 ± 8.36	0.344
Sex			0.575
Male	8	9	
Female	12	19	
	**Z-value**		
Volume (mm3)	1.171		0.129
Volume (mm3)*	0.985		0.287
I_s_	0.756		0.617
I${}_{\text{s}}^{*}$	0.706		0.702
I_BA_	1.025		0.244
I${}_{\text{BA}}^{*}$	0.985		0.286
I_δ_	1.513		0.021
I_δ_*	1.561		0.015

Volume (mm3)*: Discard outliers (No. 15, 17, 34, 45, 31). I_s_*: Discard outliers (No. 15, 31, 34, 45). I_BA_*: Discard outliers (No. 34). I_δ_*: Discard outliers (No. 5, 6, 29, 34).

**Table 3:** Statistical results of parameters related to semi-transparent and non-translucent aneurysms in the unruptured group.

	**Group B (*n* = 17)**	**Group C (*n* = 11)**	***P*-value**
Age (years)	57.18 ± 8.70	57.91 ± 8.19	0.826
Sex			0.507
Male	5	4	
Female	12	7	
	**Z-value**		
Volume (mm3)	1.645		0.009
Volume (mm3)*	1.664		0.008
I_s_	1.258		0.085
I${}_{\text{s}}^{*}$	1.333		0.057
I_BA_	1.645		0.009
I${}_{\text{BA}}^{*}$	1.579		0.014
I_δ_	1.893		0.002
I_δ_*	1.832		0.002

Volume (mm3)*: Discard outliers (No. 15, 17, 34, 45, 31). I_s_*: Discard outliers (No. 31, 45). I_BA_*: Discard outliers (No. 6, 34, 45). I_δ_*:Discard outliers (No. 6, 34).

There were no significant differences in age and gender between the ruptured aneurysm group (Group A, *n* = 20) and the unruptured aneurysm groups (Groups B + C, *n* = 28) (age: 54.95 ± 9.78 vs. 57.46 ± 8.36, *P* = 0.344, gender: *P* = 0.575). In the unruptured aneurysm groups, there were no significant differences in age and gender between the translucent group (Group B, *n* = 17) and the non-translucent (Group C, *n* = 11) (age: 57.18 ± 8.70 vs. 57.91 ± 8.19, *P* = 0.826, gender: *P* = 0.507).

### Relationship of translucent area or rupture point with isolines

**Figure 3:**
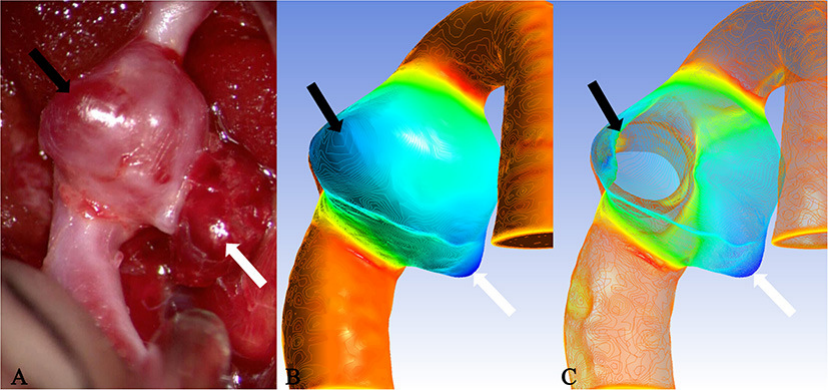
Case 9: The ruptured aneurysm in the middle cerebral artery. It was usually difficult to predict the rupture point. When the tissue adjacent to the aneurysm is not properly pulled during the operation, it is easy to cause the aneurysm to rupture and bleed again. After Polyflow simulation, the rupture point of the aneurysm could be predicted before the operation or targeted during the operation, and the operation risk would be reduced. **(A)** The white arrow pointed the location of the rupture point. The surface was covered with a fibrin cap and the area indicated by the black arrow was a translucent area, which was corresponded, respectively, to the deeply stained parts in **(B)** (black arrow and white arrow). When many areas with similar deep staining appeared on the model surface, the possible position of the rupture point could be determined through the thickness value of each extreme point in **(C)** (the white arrow indicated the thickness of the extreme point which was thinner than that of the area indicated by the black arrow).

**Figure 4:**
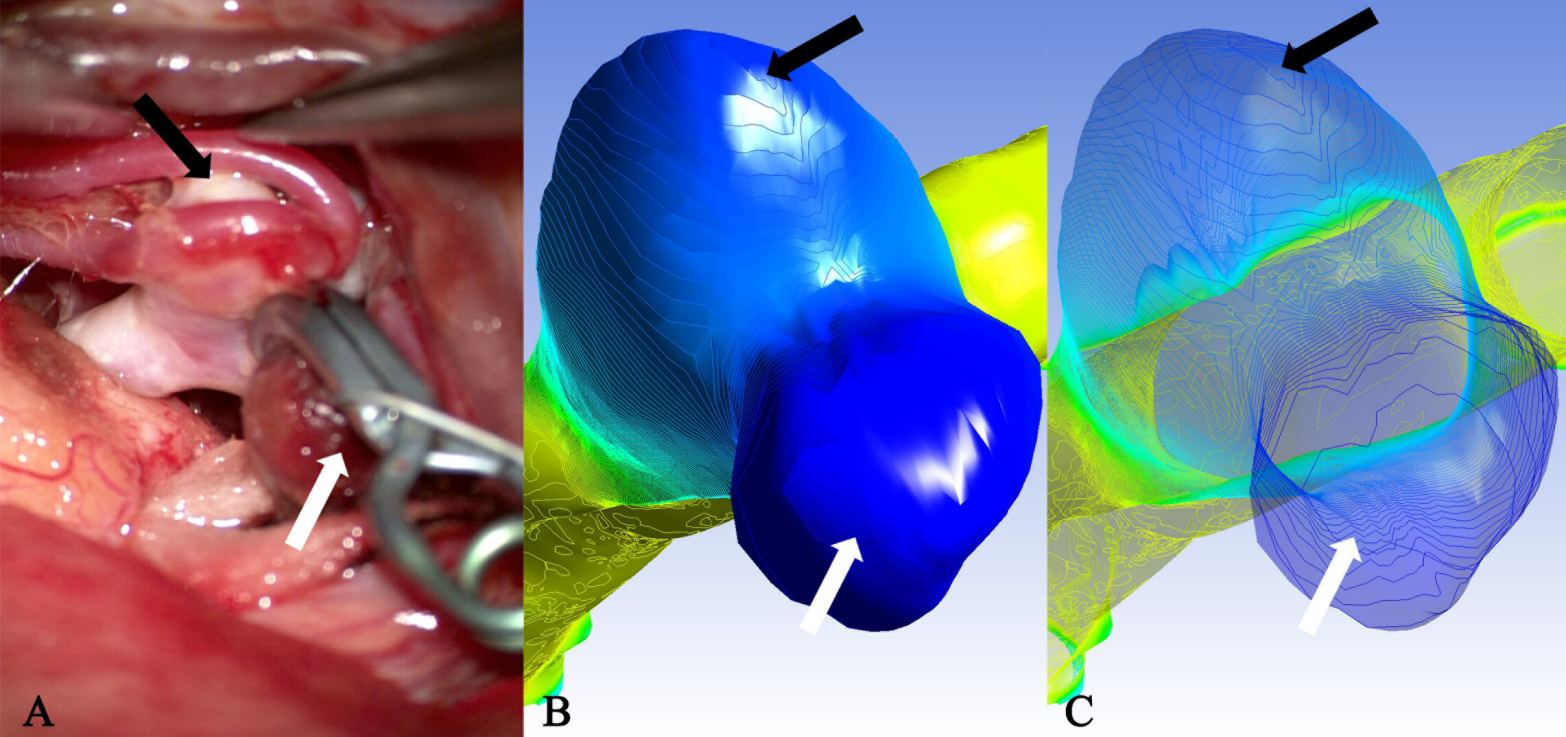
Case 2: A ruptured aneurysm in the middle cerebral artery. **(A)** The aneurysm with a bleb had been cliped. The white arrow pointed the rupture point of the aneurysm, which was completely consistent with the deeply stained part in **(B)** as indicated by the white arrow (the darker area next to it was a shadow) and the minimum extreme point in **(C)** as indicated by the white arrow. The other extreme point (black arrow) in **(C)** and the lightly deep staining area (black arrow) in **(B)** failed to show the existence of a translucent area in the corresponding part (black arrow) on the surface of the real aneurysm.

**Figure 5:**
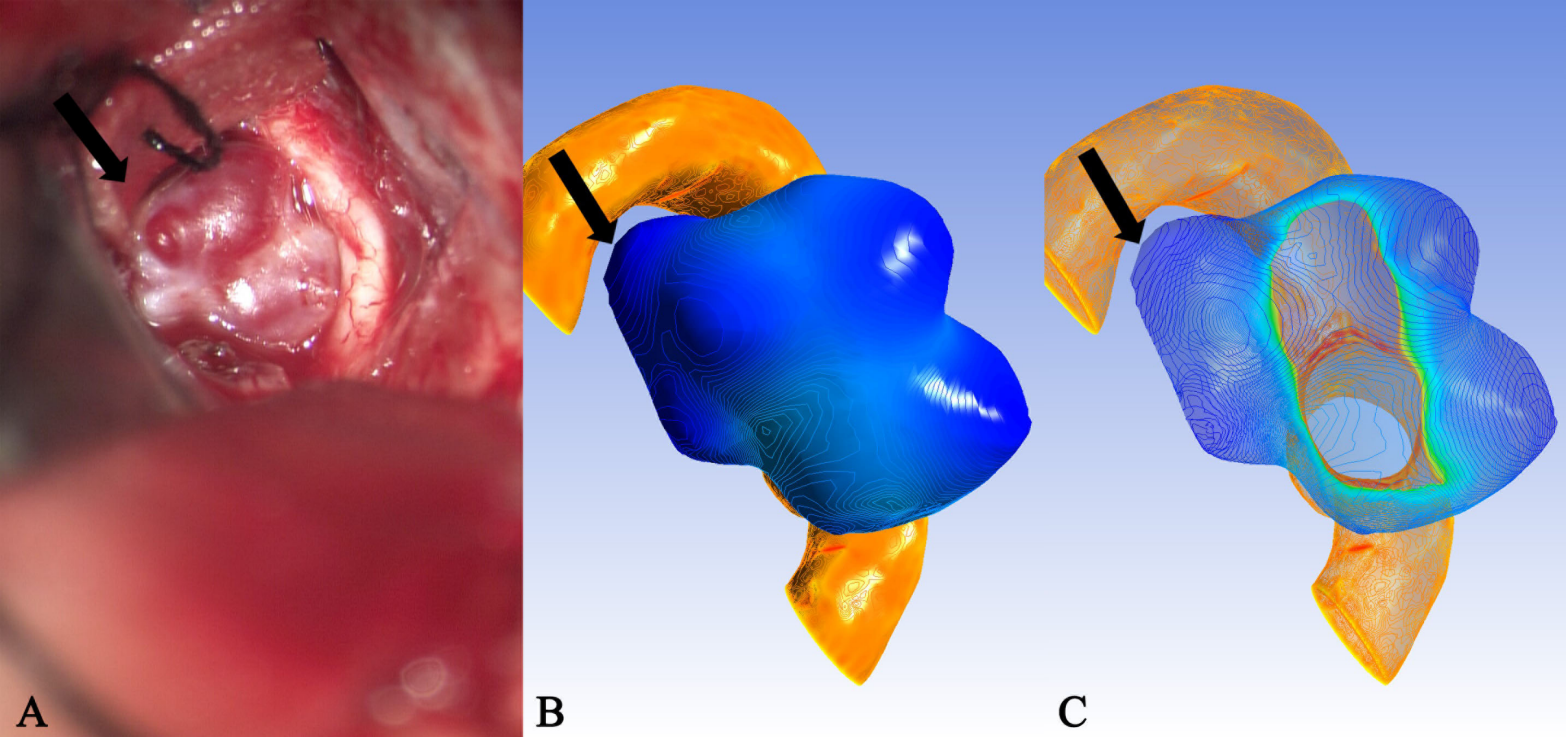
Case 35: An unruptured anterior communicating aneurysm. **(A)** There existed many translucent areas on the surface of a aneurysm entity. They all corresponded one-to-one with the deeply stained areas of the cloud map in **(B)** and the extreme points of **(C)**, but because of the limitation in the surgical visual field, some extreme points were located in the blind area of the surgical field [**(A)**, black arrow], therefore, they failed to be observed and confirmed.

**Figure 6:**
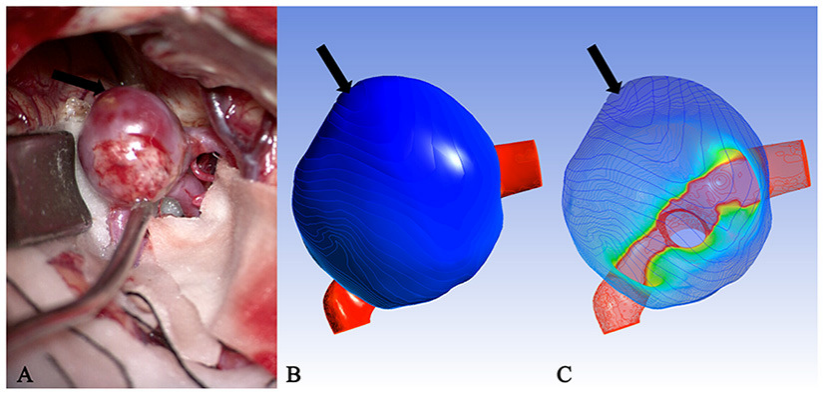
Case 15: **(A)** An unruptured aneurysm in the middle cerebral artery. The volume was listed as “outlier.” **(B)** The cloud image was uniformly stained. **(C)** The isolines near the minimum extreme point were arranged regularly and evenly spaced. It was speculated that this was also one of the reasons why some aneurysms did not rupture despite the large volume.

It is well known that isolines are closed curves. These curves are arranged in a ring shape, and the thickness corresponding to the central area surrounded by them is the extreme thickness point of the area; there may be many extreme points on the surface of a model (Figure 3). The results of the present study revealed that, in all cases, the rupture point or translucent area on the surface of the real aneurysm also had extreme thickness points in the corresponding areas on the model surface. However, the areas with extreme thickness points were not necessarily translucent areas or rupture points (Figure 4). In addition, no translucent areas or rupture points were found at the locations of the real aneurysm corresponding to the areas without extreme points on the model's surface. Due to the limitation in the visual surgical field, extreme points on the other side of some aneurysm models could not be observed and confirmed (Figure 5). Fortunately, the minimum extreme points of thickness in all model surfaces appeared within the visible range of the operative field. The distribution of isolines near the minimum extreme point of thickness also seemed to have the following characteristics: in a ruptured aneurysm or an aneurysm with a rupture tendency, the isoline intervals near the minimum extreme point were gradually sparse. In the aneurysms that tend to be stable, the isoline intervals near the minimum extreme point were regular (Figure 6).

### Geometry and parameters of aneurysms

Unruptured aneurysms seemed to have a higher coefficient of volume variation than ruptured aneurysms. We speculated that the main reason for this phenomenon might be that most cases corresponding to deviated parameters were included in the unruptured group. There was no significant difference in the volume between the ruptured and unruptured groups (Group A vs. Groups B + C) (Z = 1.171, *P* = 0.129). When the statistical analysis was carried out again with the exclusion of the outliers (No. 15, 17, 31, 34, 45), the results revealed no significant difference between the two groups (Z = 0.985, *P* = 0.287). In the patients with unruptured aneurysms, the difference was statistically significant in the volume of aneurysms between the translucent group (Group B) and non-translucent group (Group C) (z = 1.645, *P* = 0.009). When the statistical analysis was carried out again with the exclusion of the outliers (No. 15, 17, 31, 34, 45), the results were the same (Z = 1.664, *P* = 0.008). There were no significant differences in the multiple (I_s_) area increases between the ruptured and unruptured aneurysms (Z = 0.756, *P* = 0.617). Following the exclusion of the outliers (No. 15, 31, 34, 45), the results were the same (Z = 0.706, *P* = 0.702). There was no significant difference in the I_s_ of unruptured aneurysms between the translucent group (Group B) and non-translucent group (Group C) (Z = 1.258, *P* = 0.085). After excluding the outliers (No. 31, 45), the results were the same (Z = 1.333, *P* = 0.057). However, the results of the present study revealed that, during the process of aneurysm growth to rupture, there was a downward trend in the correlation between the I_S_ and volume (Group C, r = 0.900, *P* = 0.000; Group B, r = 0.745, *P* = 0.001; Group A, r = 0.241, *P* = 0.307).

### Computational fluid dynamics results

After conducting a simulated blow molding of Model B, the outer surface was fully contacted and fitted with the inner surface of Model A, forming to the shape of Model A. The thickness of Model B decreased from the aneurysm neck to the aneurysm dome. In the present study, the ratio of the initial thickness (δ_B_) of the aneurysm wall before blow molding in Model B to the thickness of extreme point δ_B_' was defined as I_BA_ to describe the magnification of the aneurysm wall thinning. There was no significant difference in I_BA_ between ruptured and unruptured aneurysms (2Z = 1.025, *P* = 0.244), and after excluding the outliers (No. 34), the results were the same (Z = 0.985 *P* = 0.286). The difference was statistically significant in I_BA_ between Groups B and C (Z = 1.645, *P* = 0.009). However, after excluding the outliers (No. 6, 34, 45), the results were the same (Z = 1.579, *P* = 0.014).

It is important to note a statistically significant difference in the coefficient of inhomogeneity I_δ_ between ruptured and unruptured aneurysms (Z = 1.513, *P* = 0.021). After excluding the outliers (No. 5, 6, 29, 34), the difference was statistically significant (Z = 1.561, *P* = 0.015). The difference in I_δ_ between Groups B and C was also statistically significant (Z = 1.893, *P* = 0.002), and after excluding the outliers, the difference remained statistically significant (Z = 1.832, *P* = 0.002).

### Multivariable analysis of the receiver operating characteristic curve

**Figure 7:**
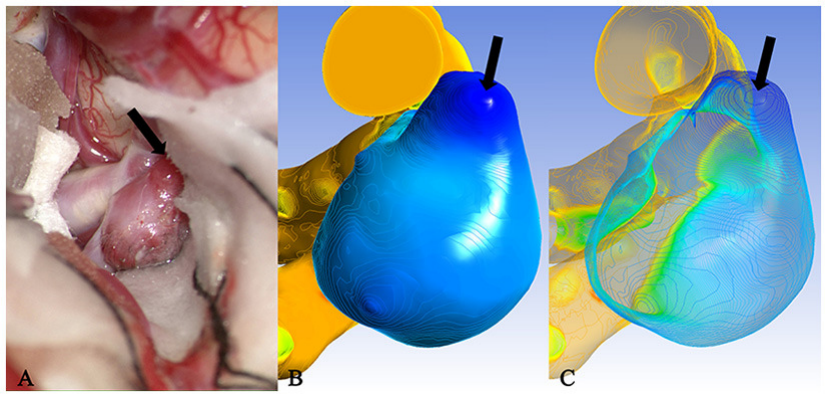
Case 46: An unruptured aneurysm in the middle cerebral artery. The translucent area (black arrow) of the surface in **(A)** corresponded to the deeply stained area (black arrow) of the cloud map in **(B)** and the minimum extreme point in **(C)**. I_δ_ of the minimum extreme point was between the optimal critical value of rupture and translucency. This was the most likely rupture point.

The area under the receiver operating characteristic curve (AUC) of the inhomogeneity coefficient I_δ_ for predicting the rupture risk of the aneurysm was 0.731 (95% CI: 0.624–0.910, *P* = 0.003) when the optimal critical value was set at 4.3991, the maximum Youden's index was 0.462, the sensitivity was 0.5, and specificity was 0.962. The AUC for predicting whether the unruptured aneurysm was translucent was 0.927 (95% CI: 0.829–1.000, *P* < 0.001) when the optimal critical value was set at 2.0753, the maximum Youden's index was 0.727, with a sensitivity of 1.000, and specificity of 0.727. The AUC of I_BA_ for predicting whether the unruptured aneurysm was translucent was 0.812 (95% CI: 0.622–1.000, *P* = 0.009. When the optimal critical value was set at 6.4424, the maximum Youden's index was 0.636, with a sensitivity of 1.000, and specificity of 0.636 (Figure 7).

## Discussion

It was concluded in the present study that the degree of uneven distribution of aneurysm wall thickness was a key factor affecting aneurysm rupture.

SAH caused by an aneurysm rupture has a high disability and mortality rate. Therefore, early detection and appropriate treatment of aneurysms are necessary to prevent SAH. Although there have been many reports on predicting the rupture risk of aneurysms, it is still unclear what state the aneurysm wall is in when it is most likely to rupture. Many researchers consider that the thickness of the aneurysm wall is associated with the rupture risk. Evaluating the characteristics of the aneurysm wall *in vitro* is one of the current difficulties. The natural history of aneurysms includes three primary stages: occurrence, growth, and stabilization or rupture. At first, mechanical tension interacts with bioremediation and is in dynamic equilibrium. Once this dynamic balance is broken, the aneurysm will enter the growth stage until the wall can no longer adapt to the tension generated, finally leading to a rupture.

Some aneurysms contain plaques and mural thrombi in the inner wall. Since DSA only shows the characteristics of the inner surface of the aneurysm, inconsistency may occur between the contour of the aneurysm model and the actual contour of the real aneurysms in the operation. However, this would not affect the results of the present study. In the present study, the convex parts on the surface of the aneurysm model did not necessarily have extreme points, and all the rupture points or translucent areas observed during the operation corresponded to the extreme points on the model.

Studies reported that ruptured aneurysms were usually irregular in shape. The convex part of the aneurysm surface is thinner and prone to rupture (8–10). Previously, researchers have tried to describe the morphology of aneurysms and the degree of irregularity in various ways, finding the relationship between each measured parameter and the risk of aneurysm rupture. But these parameters are primarily based on two-dimensional (2D) measurements, which may yield different measurements with some error when the models are in different locations in space or when the measurement is conducted by different individuals. The concept proposed by Converse Hull transforms the parameters that predict the rupture risk of the aneurysm from 2D to 3D and quantifies the description of the morphology of the aneurysm (11). However, no matter what measurement method is employed, at present, the aneurysm neck is generally regarded as a plane or a 2D line segment. We believe that aneurysms should be defined as curved surfaces, and the curvature of the aneurysm neck may affect the measurement results of the aneurysm volume and the changing rate of the area, as well as the thickness of the simulated wall. To eliminate this effect, the aneurysm neck was restored to a curved surface in the present study, and the model was processed by the interpolation method to homogenize the removal process of aneurysms, reducing the likelihood of error by minimizing the manual operation process.

The results of the present study revealed that, among the three groups of data, the correlation between I_S_ and volume showed a downward trend. The interpretation could be that in the early stage of aneurysm growth, the surface area and volume might change synchronously, while at the end stage of growth, this change process might become asynchronous, indirectly producing more irregular shapes of aneurysms. This was consistent with the research findings proposed by Dhar et al. (11) and Rajabzadeh-Oghaz et al. (12). There was an independent correlation between the undulation index (UI) and the risk of aneurysm ruptures.

In recent years, CFD has become a research hotspot in predicting the rupture risk of IAs, in which hemodynamics is investigated the most. However, the study of hemodynamics requires consideration of complex parameters, including blood properties, inertia, inlet and outlet pressures, and cardiac cycles, and there is no uniform standard for the setting of boundary conditions. The final calculation results, such as wall shear stress and oscillatory shear index, are easily affected by many factors. Therefore, the prediction of aneurysm rupture risks based on the hemodynamics research method is still controversial.

The rupture risk of aneurysms was predicted from another perspective in the present study based on fluid–structure interaction. Unlike Fluent, Polyflow has a simple execution process and can obtain the process of blow molding, stretching, and thickness change of the aneurysm wall without introducing complex parameters. Since the initial thickness of the vessel wall is offset in the latter calculation, the calculation results in the present study were presented in multiples. Therefore, the geometric details of the model played a decisive role in the calculation results. The thickness of the aneurysm wall does not change in as orderly a manner as balloon inflation. Due to the existence of the repair effect, the real thickness of the aneurysm wall may be thicker than the simulated thickness. Although the results of the present study failed to directly reflect the true thickness of the aneurysm wall, these results could indicate the distribution of aneurysm wall thickness to a certain extent and become one of the parameters by which to predict the rupture risk of an aneurysm.

A previous study reported that it was not true that the larger an aneurysm was, the more likely it was to rupture, which had been generally accepted. The rupture of aneurysms seems to be related to a specific onset location. For example, aneurysms that occur in the anterior or posterior communicating artery are more likely to rupture than aneurysms located in the internal carotid artery (13, 14). It is speculated that this may be related to the diameter of the parent artery. When the aneurysm volume was similar, the smaller the diameter of the parent artery, the greater the I_S_ was, and Is was inversely proportional to I_δ_, which seemed to contradict the above conclusion. However, the present study revealed that when I_S_ was larger, I_BA_ decreased significantly, and the final value of I_δ_ was larger, indicating that the aneurysm was more likely to rupture, which was consistent with the conclusions reported in the above literature. In addition, the change in the aneurysm neck size also affects the change of the I_S_. With the same volume, the narrower the aneurysm neck is, the greater I_S_ becomes. After CFD simulation, aneurysms with a higher inhomogeneity coefficient I_δ_ indicated a higher rupture risk of the narrow neck aneurysms. This was consistent with the research conclusions of Wan et al. (15).

When the thickness change rate I_BA_ of the minimum extreme point is the same, the area change rate I_S_ can indirectly reflect whether the aneurysm's shape is regular. When the I_S_ is smaller, it means that there must be some areas on the surface of the aneurysm that are very thin so that the same I_BA_ can be obtained, i.e., there may be one or more bulges on the surface of the aneurysm that are significantly thinner, especially in the case of an aneurysm with bleb. Therefore, the possibility of aneurysm rupture should be considered. The greater the I_S_ value, the more regular the morphology of the aneurysm and the more uniform the thickness distribution of the aneurysm wall; together with the self-repairing effect of the aneurysm wall, it can be presumed that the risk of rupture of this type of aneurysm is lower than the former.

In all cases, the highest volume outlier was 7,410.21 mm^3^, which also corresponded to the highest area ratio outlier of 17.61. In the present study, when cases with outliers in volume were reviewed, the result revealed that the clinical symptoms of these patients were mostly chronic space-occupying manifestations, such as a progressive headache. Furthermore, the actual volume of aneurysms observed during the operation was larger, the surface was smoother than the model, the vasa vasorum of the aneurysm wall was more developed and mostly had mural thrombosis, and some had calcification of the aneurysm wall. Thus, it was speculated that, during the growth of such aneurysms, the wall would be repeatedly inflamed and repaired at the same time. During the slow growth of aneurysms, the volume increases, and the distribution of the wall thickness tends to be uniform and thickened. Therefore, no rupture occurs. In those patients with outliers in geometric parameters, the final calculated I_δ_ values could typically reflect the current situation of aneurysms, such as whether they were ruptured and translucent. Therefore, we considered that outliers of geometric parameters did not seem to affect obtaining the I_δ_ value with reference significance. I_δ_ also has outliers that mainly existed in the unruptured translucent aneurysm group. For patients with these I_δ_ outliers, the irregular aneurysm shape could be observed during the operation, and the region where the minimum extreme point was located had become translucent. In theory, ruptures had occurred, but in fact, the weakest parts of the aneurysms were wrapped by bony structures, membranous structures, or brain tissue, which played a protective factor and delayed the rupture to a certain extent.

At present, when predicting the rupture risk of an aneurysm through morphology or CFD, the common complicated problem is that when it ruptures, the size and shape may change, and the occurrence of spasms of the parent artery may have a certain impact on the research results. The parameters directly affected by morphology changes are I_S_ and volume, while the parameters directly affected by the spasms of parent vessels are I_S_ and I_BA_. The changes of these parameters ultimately have an impact on I_δ_.

As a CFD module, Polyflow could predict the location of the rupture point of an aneurysm and determine whether it was translucent. When translucent areas appear on the surface of aneurysms, they might rupture in the near future. The analysis of a combination of CFD results and morphology parameters would have a higher value in predicting the rupture risk of an aneurysm.

## Conclusion

The Polyflow module of CFD could be used as an effective tool to predict the rupture point or detect a translucent area of an aneurysm's surface. Inhomogeneity coefficient I_δ_ would be an effective parameter to predict the rupture risk of an aneurysm, which might have some guiding significance for clinical diagnosis and treatment.

## Data availability statement

The original contributions presented in the study are included in the article/supplementary material, further inquiries can be directed to the corresponding author.

## Ethics statement

The studies involving human participants were reviewed and approved by Xuanwu Hospital. The patients/participants provided their written informed consent to participate in this study.

## Author contributions

Conception and design of the research and writing of the manuscript: X-xF and H-qZ. Acquisition of data, analysis and interpretation of the data, and critical revision of the manuscript for intellectual content: X-xF, J-wG, CH, PH, L-yS, and H-qZ. Statistical analysis: X-xF and J-wG. Obtaining financing: H-qZ. All authors have read and approved the final draft.
